# Discontinuing Hepatitis Activity Reduced Hepatocellular Carcinoma Recurrence after Primary Curative Therapy

**DOI:** 10.3390/jpm13030397

**Published:** 2023-02-24

**Authors:** Chern-Horng Lee, Chien-Heng Shen, Cho-Li Yen, Tzung-Hai Yen, Sen-Yung Hsieh

**Affiliations:** 1 Division of General Internal Medicine and Geriatrics, Chang Gung Memorial Hospital, Linkou Branch, Taoyuan 333, Taiwan; 2 Department of Gastroenterology and Hepatology, Chang Gung Memorial Hospital, Chiayi Branch, Chiayi 613, Taiwan; 3 Department of Gastroenterology and Hepatology, Chang Gung Memorial Hospital, Keelung Branch, Keelung 204, Taiwan; 4 College of Medicine, Chang Gung University, Taoyuan 333, Taiwan; 5 Department of Nephrology, Chang Gung Memorial Hospital, Linkou Branch, Taoyuan 333, Taiwan; 6 Department of Gastroenterology and Hepatology, Chang Gung Memorial Hospital, Linkou Branch, Taoyuan 333, Taiwan

**Keywords:** hepatocellular carcinoma, tumor recurrence, risk factors, hepatitis treatments

## Abstract

Background: Hepatocellular carcinoma (HCC) tends to recur after curative treatment. This study aimed to identify the clinical factors associated with HCC recurrence after initial curative therapy. Methods: We retrospectively included patients with early stage HCC Barcelona Clinic Liver Cancer (BCLC) stages 0 and A who received curative surgical resection or local ablation at three different Chang Gung Memorial Hospitals in Taiwan (527 patients from Linkou, 150 patients from Keelung, and 127 patients from Chiayi) from 2000 to 2009. Pretreatment clinical data were subjected to univariate and multivariate logistic analyses to identify the risk factors for HCC recurrence within five years after the primary curative treatment. Recurrence and survival rates were assessed using Kaplan–Meier curves and log-rank tests. Results: Patients with a history of nucleoside analog or peg-interferon treatment for hepatitis B or hepatitis C infection had lower HCC recurrence rates than did those without such treatment. By contrast, alcohol drinking habits (*p* = 0.0049, hazard ratio (HR): 1.508, 95%CI: 1.133–2.009), a platelet count of < 14 × 10^4^/μL (*p* = 0.003, HR: 1.533, 95%CI: 1.155–2.035), and a serum alanine aminotransferase level > 40 U/L (*p* = 0.0450, HR: 1.305, 95%CI: 1.006–1.694) were independent risk factors for HCC recurrence. The five-year HCC recurrence rates did not differ between patients who received either local radiofrequency ablation or surgical resection at BCLC stages 0 and A. Conclusions: Factors contributing to persistent hepatitis activity and advanced fibrosis precipitate tumor recurrence. Active intervention to discontinue liver injury or hepatitis could reduce HCC recurrence.

## 1. Introduction

Post-treatment tumor recurrence is one of the most challenging issues to overcome when working toward the long-term survival of patients with hepatocellular carcinoma (HCC). HCC is the fourth most common cause of cancer-related death worldwide [1]. Despite considerable improvements in HCC detection and treatment in the past two decades, the overall survival rate for HCC remains unsatisfactory. One of the main causes of poor clinical outcomes of HCC patients is its unusually high local and systemic recurrence rates, even after primary curative therapy. It has been reported that the recurrence rates at 1, 3, and 5 years posttherapy are approximately 22–26.3%, 36.1–62%, and 49.6–74%, respectively, in patients with Barcelona Clinic Liver Cancer (BCLC) stage 0-B [2,3,4,5,6]. HCC recurrence is associated with poor clinical outcomes with a median survival time as short as 68.5 weeks after radical surgical resection [7]. Indeed, clinical factors associated with the recurrence of HCC have been extensively studied. Zhou L. et al. reported that ALT and AST were associated with overall recurrence and post-recurrence survival in univariate analyses; according to multivariate tests, AST was marginally significant for early recurrence and post-recurrence survival [8]. Multivariate analysis indicated that Tenofovir (nucleoside analogue) could decrease the recurrence of HCC in chronic hepatitis B patients after liver resection (hazard ratio, 0.35; 95% confidence interval range, 0.33–0.84, *p* < 0.05) [9]. Neither beta blocker usage nor serum ALT levels predict HCC recurrences following non-metastatic HCC in patients who have undergone surgical resections and/or radiofrequency ablation (RFA) [10]. Multivariate analysis showed that, in patients with initial non-advanced HCC with HCV who underwent RFA, low albumin levels and high AST levels were independent predictive factors for distant recurrence [11]. HCC recurrence was less commonly observed in patients with chronic hepatitis C who received curative treatment for HCC, compared with those patients who received noncurative treatment in the form of direct-acting antiviral (DAA) therapy (*p* = 0.007) [12]. However, to improve long-term outcomes, it is crucial to conduct a comprehensive analysis of the clinical, laboratory, and pathological confounders, using a sizable cohort to identify clinically applicable factors in order to identify the subgroup of patients at an early stage of HCC but at high risk of tumor recurrence.

It has been reported that multiple tumors, gene expression signatures associated with aggressive tumor behavior, poor tumor differentiation, vascular invasion, macrosatellites, and microsatellites are risk factors for HCC recurrence after liver surgery or a transplant for HCC [13,14]. Compound killer cell immunoglobulin-like receptor–human leukocyte antigen genotypes with multiplicity are also predictive factors for HCC recurrence [15]. However, these risk factors depend on tumor tissues that are not available before tumor resection and cannot be generalized to most patients.

Accordingly, we conducted our study to identify the pretreatment clinical factors that can predict HCC recurrence before the primary treatment. Our study aims to contribute to reducing post-treatment tumor recurrence, and it identifies for adjuvant therapies a subgroup of patients with early-stage HCC who have a high risk of post-treatment recurrence.

## 2. Materials and Methods

### 2.1. Clinicopathological Characteristics of the Studied Patients

We retrospectively included consecutive patients with HCC admitted to any of the three branches of Chang Gung Memorial Hospitals in Linkou, Keelung, and Chiayi between 2000 and 2009. 

**Figure 1:**
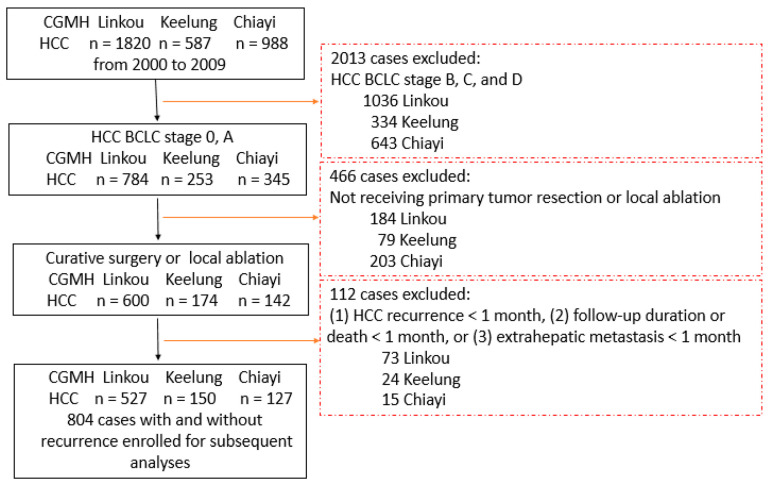
Flow chart of patient selection. CGMH, Chang Gung Memorial Hospital; HCC, hepatocellular carcinoma.

We initially enrolled 3395 patients with HCC who were diagnosed and received treatment at three Chang Gung Memorial Hospitals located in different regions of Taiwan (1820 patients from Linkou, 587 patients from Keelung, and 988 patients from Chiayi) from 2000 to 2009. After applying the exclusion criteria, a total of 804 patients (527, 150, and 127 from Linkou, Keelung, and Chiayi, respectively) were included in the subsequent analyses (Figure 1).

We reviewed patients’ demographic and clinical data, including sex, age, the presence of liver cirrhosis, alcohol use, the number of tumors, the largest tumor size, the presence of ascites, and laboratory data (i.e., serum α-fetoprotein (AFP), albumin, bilirubin, creatinine, aspartate aminotransferase [AST], alanine aminotransferase [ALT], prothrombin time [PT], and blood cell count), the date of the primary tumor resection, the date of local recurrence, and the date of the last follow-up or HCC-related death. Platelet (1000/μL) counts and serum alanine aminotransferase (U/L) were measured using the automated cell counter and enzymatic methods, respectively. The clinical outcomes were assessed for up to 5 years after the primary treatment.

The HCC stage was defined according to the BCLC staging system [16]. We excluded patients at the late tumor stage (BCLC stage > A), those with tumor recurrence within one month, and those who failed to attend follow-up or who died within one month of the initial treatment.

During the initial treatment, curative treatment was defined as complete tumor resection or local radiofrequency ablation (RFA). The date of HCC recurrence was determined as the date when a newly developed liver tumor was discovered after the curative treatment [17]. Intrahepatic HCC recurrence was defined as a newly developed tumor, not a primary site lesion. Time to recurrence was defined as the duration from the curative therapy to the date of the first detection of tumor recurrence.

The primary study was ended at one of the following points: the detection of HCC recurrence through an examination during the follow-up period, the patient’s death, failure to follow up, or the last patient visit.

### 2.2. Diagnosis of HCC

HCC was diagnosed based on dynamic imaging studies and biopsy procedures, in accordance with the Asia-Pacific clinical practice guidelines [18]. A biopsy was performed only when an atypical or equivocal image of HCC was not obtained.

### 2.3. Follow-Up Studies

All eligible cases were followed up every two to three months for up to five years and three to six months after that. At each follow-up visit, a complete history was taken, a physical examination was performed, a blood sample was drawn for a serum AFP assay and liver biochemistry tests, and tumor recurrence was monitored using ultrasonography and a chest X-ray. When HCC recurrence or metastasis was suspected, computed tomography, hepatic arteriography, or magnetic resonance imaging was performed for confirmation.

### 2.4. Statistical Analysis

We used Student’s *t*-test to compare the continuous variables. We also used the chi-square test for categorical variables to identify risk factors for HCC. The Cox proportional hazards model was used to compare relative risk estimates between those with and without recurrence. Recurrence and survival rates were assessed using the Kaplan–Meier method. Comparisons of survival durations were made using the log-rank test. *p* < 0.05 (two-tailed) was considered statistically significant. Data were statistically analyzed using Statistical Analysis System (SAS) software (SAS Institute, Inc., Cary, NC, USA).

## 3. Results

### 3.1. Clinical Characteristics of the Patients

We compared the baseline clinical characteristics of patients with and without HCC recurrence over five years after the initial curative therapies. Parameter factors with significant difference include alcohol drinking (*p* = 0.023), HBV (+) (*p* = 0.025), HCV (+) (*p* = 0.005), albumin < 3.0 g/dl (*p* = 0.0036), total bilirubin > 3.0 mg/dL (*p* = 0.0180), AST > 40 U/L (*p* = 0.029), ALT > 40 U/L (*p* = 0.003), a platelet count < 14 × 10^4^/μL, nucleoside analogue (*p* = 0.011), and nucleoside analogue or interferon (*p* = 0.005) (Table 1).

**Figure 2:**
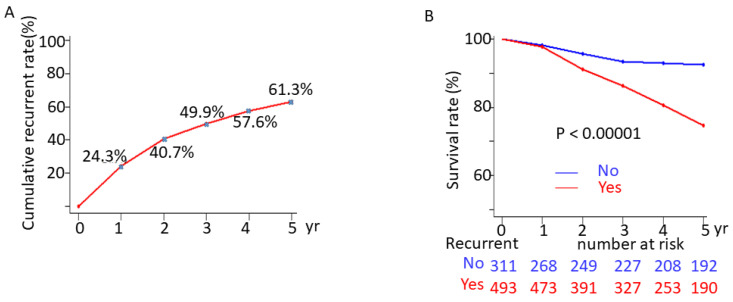
(**A**) Cumulative recurrent incidence of HCC post-curative treatment. (**B**) Survival rates of HCC patients after curative treatment with and without recurrence.

The median follow-up duration was 9.28 years, and the median time to recurrence was 1.998 years. The cumulative overall recurrence rates at 1, 2, 3, 4, and 5 years were 196 (24.3%), 327 (40.7%), 401 (49.9%), 463 (57.6%), and 493 (61.3%) cases, respectively. (Figure 2A) The survival rates in patients with recurrence versus those without recurrence were 97.77% versus 97.75%, 87.63% versus 92.92%, and 73.63% versus 91.64% at 1, 3, and 5 years, respectively (Figure 2B). 

### 3.2. Risk Factor Selection

**Figure 3:**
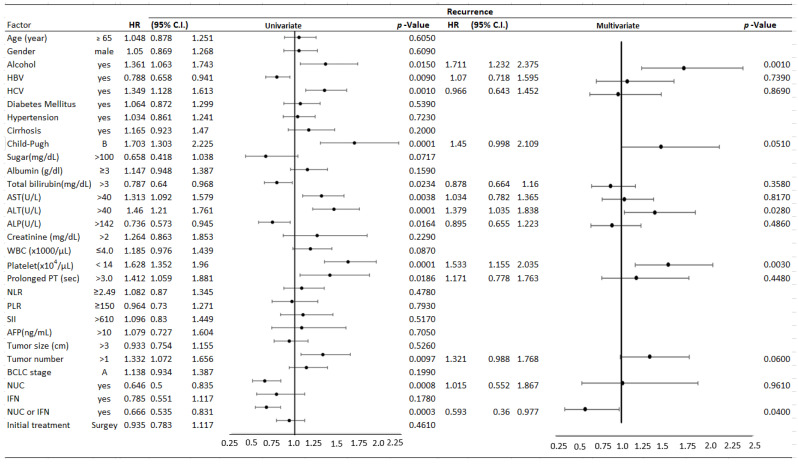
Univariate and multivariate stratified analyses for early-stage hepatocellular carcinoma (HCC), based on Barcelona Clinic Liver Cancer (BCLC) staging, with and without tumor recurrence after the initial curative treatment (forest plot). AFP, α-fetoprotein; ALP, Alkaline Phosphatase; ALT, alanine aminotransferase; AST, aspartate aminotransferase; CI, confidence interval; CBC, counts of blood cells; NLR, neutrophil to lymphocyte ratio; PLR, platelet to lymphocyte ratio; PT, prothrombin time; HBV, hepatitis B virus surface antigen; HCV, antibodies to hepatitis C virus; HR, hazard ratio; and SII, systemic immune-inflammation index.

We pooled all patients from the three branches of Chang Gung Memorial Hospital for analysis. In our univariate analysis, alcohol drinking habits (*p* = 0.015), HBV (*p*= 0.009), HCV (*p* = 0.001), Child–Pugh class B (*p* < 0.001), AST level > 40 U/L (*p* = 0.0038, ALT level > 40 U/L (*p* < 0.0001), total bilirubin (*p*= 0.0234), ALP (*p* = 0.0164), a pretreatment platelet count < 14 × 10^4^/µL (*p* < 0.0001), prolonged PT (*p* = 0.0186), the number of tumors (*p* = 0.0097), nucleoside analogue (NUC) (*p*= 0.0008), and nucleoside analogue (NUC) or interferon therapy (*p* = 0.0003), were significant risk factors associated with HCC recurrence (Table 2). In our multivariate analysis, alcohol drinking habits (*p* = 0.001, HR: 1.711, 95%CI: 1.232–2.375), the ALT level > 40 U/L (*p* = 0.028, HR: 1.379, 95%CI: 1.035–1.838), and platelets < 14 × 10^4^/μL (*p* =0.003, HR: 1.533, 95%CI: 1.1.155–2.035) were independent risk factors for HCC recurrence in 5 years (Table 2 and Figure 3). Nucleoside analogue or interferon could reduce HCC recurrence (*p* = 0.04, HR: 0.593, CI: 0.360–0.977). The HCC recurrence rates did not differ between patients receiving local radiofrequency ablation or surgical intervention.

### 3.3. Comparison of HCC Recurrence Rates through the Log-Rank Test

**Figure 4:**
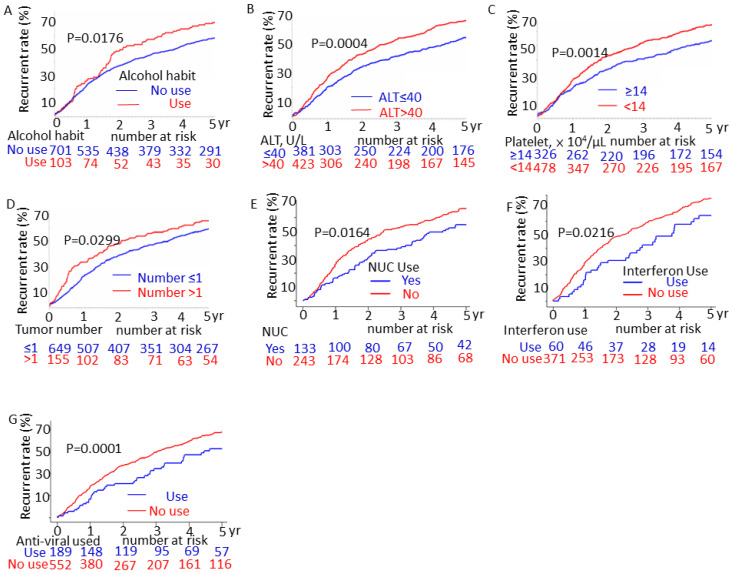
Univariate Cox regression analysis of the cumulative recurrence rates of early hepatocellular carcinoma (HCC) post-primary curative therapy (*p* values determined by the log-rank test): (**A**) alcohol habits, (**B**) alanine aminotransferase (ALT) level, (**C**) platelet count, (**D**) tumor number, (**E**) patients with hepatitis B virus (HBV) infection with and without nucleoside analog (NUC) treatment, and (**F**) patients with hepatitis C virus (HCV) infection with and without interferon treatment, (**G**) patients with and without antiviral treatments (nucleoside analog (NUC)) or interferon.

We used the log-rank test to assess five-year recurrence rates. Patients who had alcohol drinking habits (vs. no alcohol drinking habits; *p* = 0.0176; Figure 4A), an ALT level of >40 U/L (vs. <40 U/L; *p* = 0.0004; Figure 4B), or a platelet count of <14 × 10^4^/μL (vs. >14 × 10^4^/μL; *p* = 0.0014; Figure 4C) had higher five-year recurrence rates.

Patients with hepatitis B virus (HBV) infection receiving nucleoside analogs (NUC) had a lower five-year recurrence rate than did those who were not receiving nucleoside analog therapy (*p* = 0.0164; Figure 4E). Moreover, patients with hepatitis C virus (HCV) infection also had a lower likelihood of recurrence if receiving interferon treatment (*p* = 0.0216; Figure 4F). Patients receiving antiviral therapy had a lower recurrence rate (*p* = 0.0001; Figure 4G).

### 3.4. Survival Rates of Patients with HCC with and without Confounding Factors

**Figure 5:**
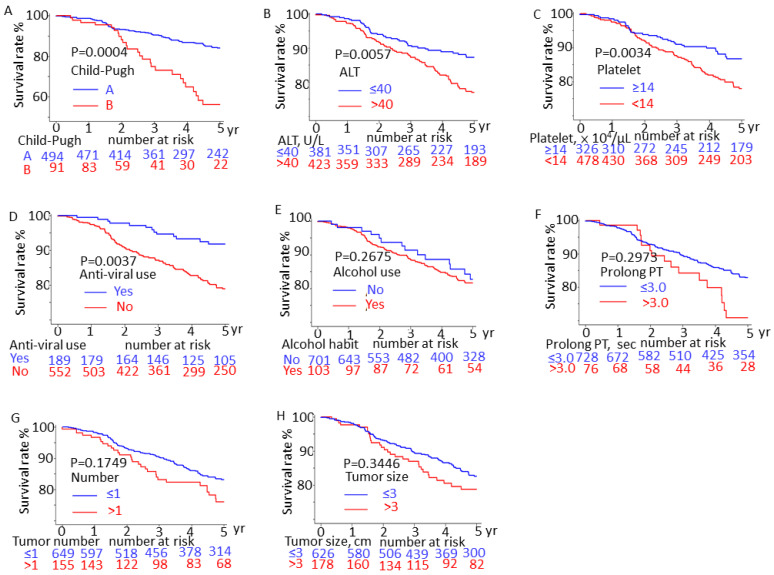
Effects of risk factors on five-year survival rates in early stage HCC (BCLC stage 0-A) with recurrence after curative treatment (log-rank test): (**A**) Child–Pugh scores, (**B**) aspartate aminotransferase (ALT) level, (**C**) platelet count, (**D**) with and without therapy antiviral agents (nucleoside analog or interferon), (**E**) alcohol habits, (**F**) prothrombin time (PT), (**G**) tumor number, and (**H**) tumor size.

We also used the log-rank test to assess survival rates. We observed that patients who had a Child–Pugh class B disease (vs. Child–Pugh class A disease; *p* = 0.00004; Figure 5A), an ALT level of >40 U/L (vs. ≤ 40 U/L; *p* = 0.0057; Figure 5B), a platelet count of <14 × 10^4^/μL (vs. ≥14 × 10^4^/μL; *p* = 0.0034; Figure 5C), or those who were not receiving antiviral therapy (vs. receiving anti-viral therapy; *p* = 0.0037; Figure 5D) exhibited significantly lower five-year survival rates.

## 4. Discussion

We identified the following risk factors associated with post-treatment HCC recurrence: alcoholic habits, an ALT level of >40 U/dL, and a platelet count of <14 × 10^4^/μL. On the other hand, anti-viral treatment with nucleoside/nucleotide analogues or interferons is a protective factor for recurrence. These findings further affirm the significance of persistent inflammation in HCC recurrence. In addition, these easy-to-assess clinical factors enable the early identification of the subgroup of HCC patients who are scheduled to receive curative therapy but who are at a high risk of HCC recurrence, facilitating the prior planning of personalized therapeutic regimens.

It is surprising that, in HCC patients at the BCLC early stage, geriatric status, gender (male), diabetes, hypertension, albumin < 3.0 (g/dl), prolonged PT > 3.0 (sec), bilirubin > 3.0 (mg/dl), creatinine > 2.0 (mg/dL), WBC ≤ 4.0 (×1000/μL), SII > 610 × 1000/μL, AFP > 10 (ng/mL), tumor size > 3 (cm), and the initial treatment mode (RFA vs. surgery) were not significantly associated with HCC recurrence. AST > 40 (U/L) was also not associated with HCC recurrence, although some papers suggest that it is a predictor of HCC recurrence [8]. One explanation for the inconsistency of our findings with others is that the cases included in this study were confined to patients who had been diagnosed (BCLC stage 0-A) and received curative therapies including RFA and tumor resection. Our findings further reinforce the importance of the early diagnosis and early treatment of HCC in the prevention of tumor recurrence and the improvement of overall survival rates. 

Alcohol is known to be detrimental to liver cells and causes inflammation. Alcoholic liver disease is characterized by simple steatosis, steatohepatitis, fibrosis, and cirrhosis leading to HCC [19]. Chronic alcohol consumption may impair autophagy and contribute to the pathogenesis of the alcoholic liver disease [20]. On the other hand, alcohol consumption generates acetaldehyde and accumulates reactive oxygen species, interfering with DNA methylation, synthesis, and repair, and leading to increased HCC susceptibility [21]. Moreover, once alcoholic cirrhosis has developed, liver carcinogenesis continues unabated even after abstinence from alcohol. Indeed, it has been reported that alcohol consumption habits in cirrhotic patients synergistically triples their risk of HCC [22]. Our finding that alcohol consumption is a significant risk factor for HCC recurrence further confirms the role of alcohol drinking in hepatocarcinogenesis and indicates the significance of immediate alcohol abstinence in reducing the incidence of HCC recurrence. Constance Marié et al. report that alcohol abstinence could be effective in controlling the progression and slowing the spread of aggressive HCC [23]. 

Previously, it has been reported that hepatitis activity (a high ALT level) is one of the main risk factors associated with HCC development in patients with chronic hepatitis B or C [24,25]. Chronic hepatitis B and chronic hepatitis C have common mechanisms of hepatocarcinogenesis, including persistent liver inflammation, immune-mediated damage, and the disruption of cell cycle pathways [26]. Herein, we found that patients with a higher ALT level had a higher recurrence rate, while those who received anti-viral therapies had a lower recurrent rate. Together, these findings indicate the pathogenic role of persistent hepatitis in both the de novo development and recurrence of HCC. Further, in addition to immediately controlling hepatitis activity by means such as alcohol abstinence, anti-viral treatment is critically important not only for preventing primary HCC occurrence, but also for reducing the HCC recurrence rate after initially curative treatments. Indeed, previous studies have shown that the treatments for viral hepatitis B and C reduce HCC incidence by 10–40% [27,28,29]. Syed T. et al. reported on 497 HCV-infected patients who were treated with DAAs therapy, or with a combination of DAA with interferon; rates of SVR were much lower in the cirrhotic group in those who were eventually diagnosed with HCC compared to those who were not (62.5% vs. 88.94%, *p* = 0.002), respectively [30]. In this study, we found that anti-viral therapies could reduce HCC recurrence by approximately 40%.

HCC patients with a platelet count of <14 × 10^4^/μL had more frequent recurrence and worse survival than those with a platelet count of ≥14 × 10^4^/μL in BCLC early-stage HCC. Alberto Zanetto et al. found that HCC is associated with increased platelet aggregation and higher Von Willebrand factor antigen (VWF) levels in cirrhotic patients [31]. Platelets have multiple functions, and platelets and products may be associated with tumor proliferation and extrahepatic metastasis [32]. Thrombocytopenia is highly correlated with the severity of liver cirrhosis [33,34]. Patients with thrombocytopenia had an increased risk of post-resection recurrence [35], though some studies have reported primary HCC chemoprevention with daily aspirin or other antiplatelet agents. Whether aspirin or antiplatelet agents can prevent posttreatment HCC recurrence remains to be studied. However, in this study, HCC with a platelet count of <14 × 10^4^/μL indicates more advanced hepatic cirrhosis and more easily leads to the development of HCC recurrence than HCC with a platelet count of ≥14 × 10^4^/μL. Our findings support those of a previous study, in which the postresection recurrence rate of HCC was in an inverse relationship with the platelet count and could be stratified accordingly [36].

Cirrhosis increases the synergic effect of HCC development and recurrence in viral hepatitis patients [22,37,38]. Our patients in Child–Pugh class B had significantly higher recurrence incidence than those in Child–Pugh class A (*p* < 0.0001, HR: 1.703 in univariate). Multivariate analysis showed a trend of HCC recurrence (*p* = 0.051, HR: 1.45). 

As a result, identifying risk factors for HCC recurrence before treatment can help to identify subgroups of early stage HCC patients with a high risk of recurrence, enabling clinicians to design personalized treatment regimens to reduce potential HCC recurrence. As such, multimodal therapy can be considered for all those harboring the relevant pretreatment risk factors for recurrence; this therapy might include antiviral therapy and alcohol abstinence, with the aim of lowering the incidence of post-treatment HCC recurrence [39]. 

The limitations of this study include the retrospective cohort study design; the small sample size, the lack of data, and the new development treatment modalities. Large-scale prospective studies are necessary further to validation of this observation.

## 5. Conclusions

Alcoholic habits, a lack of a history of anti-viral treatments, higher serum ALT levels, and lower platelet counts are pre-treatment clinical factors associated with post-treatment tumor recurrence in patients with early-stage HCC. Our findings reaffirm the significance of the active termination of persistent liver injury or hepatitis by abstaining from drinking alcohol or by receiving anti-viral therapy in reducing the post-treatment recurrence rate of HCC. We also envision that the emergence of DAAs therapies for the eradication of HCV infection will also mitigate HCC recurrence in patients with chronic hepatitis C. 

## Figures and Tables

**Table 1:** Baseline clinical characteristics of patients with and without post-curative hepatocellular carcinoma (HCC) recurrence over five years.

	without Recurrence		with Recurrence		*p*-Value
	*n* = 311	%	*n* = 493	%	
Age (year)					
<65	165	20.52%	261	32.46%	0.975
≥65	146	18.16%	232	28.86%	
Sex (*n*, %)					
Male	107	13.31%	160	19.90%	0.591
Female	204	25.37%	333	41.42%	
Alcohol (yes)	29	3.61%	74	9.20%	0.023
HBV (yes)	161	20.02%	215	26.74%	0.025
HCV (yes)	147	18.28%	284	35.32%	0.005
Diabetes mellitus (yes)	81	10.07%	132	16.42%	0.870
Hypertension (yes)	108	13.43%	182	22.64%	0.547
Cirrhosis (yes)	243	30.22%	407	50.62%	0.141
Child–Pugh					0.070
A	189	23.51%	305	37.94%	
B	25	3.11%	67	8.33%	
Sugar >100 mg/dL	71	8.83%	123	15.30%	0.060
Albumin < 3.0 g/dl	130	16.17%	183	22.76%	0.312
Total bilirubin > 3.0 mg/dL	97	12.06%	123	15.30%	0.150
AST > 40 U/L	108	13.43%	216	26.87%	0.029
ALT > 40 U/L	140	17.41%	283	35.20%	0.003
ALP > 142 U/L	214	26.62%	305	37.94%	0.099
Creatinine > 2.0 mg/dL	16	1.99%	28	3.48%	0.946
WBC ≤4000/μL	77	9.58%	140	17.41%	0.025
Platelet < 14 × 10^4^/μL	156	19.40%	314	39.05%	<0.001
Prolonged PT 3.0–5.0 sec	23	2.86%	53	6.59%	0.262
NLR < 2.49	148	18.41%	237	29.48%	0.333
PLR < 150	195	24.25%	301	37.44%	0.438
SII > 610 × 1000/μL	38	4.73%	58	7.21%	0.963
AFP > 10 ng/mL	272	33.83%	430	53.48%	0.612
Tumor size >3 cm	75	9.33%	108	13.43%	0.490
Tumor number >1	52	6.47%	103	12.81%	0.168
BCLC 0	102	12.68%	137	17.03%	
A	209	25.99%	349	43.41%	0.307
Nucleoside analogue (yes)	65	8.08%	68	8.46%	0.011
Interferon (yes)	27	3.36%	33	4.10%	0.335
Nucleoside analogue or interferon (yes)	90	11.19%	99	12.31%	0.005
First treatment					
RFA	135	16.79%	225	27.99%	0.561
Surgery	176	21.89%	268	33.33%	

All statistical tests were two-tailed and used a type I error rate of 0.05 (*p*). AFP: α-fetoprotein; ALT: alanine aminotransferase; ALP, alkaline phosphatase; CI: confidence interval; HBV: HBsAg positive; HCV: anti-HCV antibody; INR: International Normalized Ratio; NLR: neutrophil to lymphocyte ratio; RFA: local radiofrequency ablation; PLR: platelet to lymphocyte ratio; SII: neutrophil × platelet/lymphocyte.

**Table 2:** Risk factors for hepatocellular carcinoma (HCC) recurrence according to univariate and multivariate analyses.

		Univariate Cox Model				Multivariate Cox Model			
Variable		HR	95%CI		*p*-Value	HR	95%CI		*p*-Value
Age (year)	≤65	1.000	-	-	-				
	>65	1.048	0.878	1.251	0.605				
Sex	Male	1.000	-	-	-				
	Female	1.050	0.869	1.268	0.609				
Alcohol	No	1.000	-	-	-	1.000	-	-	-
	Yes	1.361	1.063	1.743	0.015	1.711	1.232	2.375	0.001
HBV	No	1.000	-	-	-	1.000	-	-	-
	Yes	0.788	0.658	0.941	0.009	1.070	0.718	1.595	0.739
HCV	No	1.000	-	-	-	1.000	-	-	-
	Yes	1.349	1.128	1.613	0.001	0.966	0.643	1.45	0.869
Diabetes mellitus	No	1.000	-	-	-				
	Yes	1.064	0.872	1.299	0.539				
Hypertension	No	1.000	-	-	-				
	Yes	1.034	0.861	1.241	0.723				
Cirrhosis	No	1.000	-	-	-				
	Yes	1.165	0.923	1.470	0.2				
Child–Pugh score	A	1.000				1.000			
	B	1.703	1.303	2.225	<0.0001	1.450	0.998	2.109	0.051
Sugar	≤100	1.000	-	-	-				
(mg/dL)	>100	0.658	0.418	1.038	0.0717				
Albumin	<3.0	1.000	-	-	-				
(g/dl)	≥3.0	1.147	0.948	1.387	0.159				
Total bilirubin	≤3.0	1.000	-	-	-	1.000	-	-	-
(mg/dl)	>3.0	0.787	0.640	0.968	0.0234	0.878	0.664	1.160	0.358
AST	≤40	1.000	-	-	-	1.000	-	-	-
(U/L)	>40	1.313	1.092	1.579	0.0038	1.034	0.782	1.365	0.817
ALT	≤40	1.000	-	-	-	1.000	-	-	-
(U/L)	>40	1.46	1.21	1.761	<0.0001	1.379	1.035	1.838	0.028
ALP	≤142	1.000	-	-	-	1.000	-	-	-
(U/L)	>142	0.736	0.573	0.945	0.0164	0.895	0.655	1.223	0.486
Creatinine	≤2.0	1.000	-	-	-				
(mg/dL)	>2.0	1.264	0.863	1.853	0.229				
WBC	>4.0	1.000	-	-	-				
(×1000/μL)	≤4.0	1.185	0.976	1.439	0.087				
Platelet (× 10^4^/μL)	≥14	1.000	-	-	-	1.000	-	-	-
	<14	1.628	1.352	1.960	<0.0001	1.533	1.155	2.035	0.003
Prolonged PT	≤3.0	1.000	-	-	-	1.000	-	-	-
(sec)	>3.0	1.412	1.059	1.881	0.0186	1.171	0.778	1.763	0.448
NLR	<2.49	1.000	-	-	-				
	≥2.49	1.082	0.870	1.345	0.478				
PLR	<150	1.000	-	-	-				
	≥150	0.964	0.730	1.271	0.793				
SII	≤610	1.000							
	>610	1.096	0.830	1.449	0.517				
AFP	≤10	1.000	-	-	-				
(ng/mL)	>10	1.079	0.727	1.604	0.705				
Tumor size	≤3	1.000	-	-	-				
(cm)	>3	0.933	0.754	1.155	0.526				
Tumor number	≤1	1.000	-	-	-	1.000	-	-	-
	>1	1.332	1.072	1.656	0.0097	1.321	0.988	1.768	0.060
BCLC stage	0	1.000							
	A	1.138	0.934	1.387	0.199				
Nucleoside analogue	No	1.000				1.000			
	Yes	0.646	0.5	0.835	0.0008	1.015	0.552	1.867	0.961
Interferon	No	1.000							
	Yes	0.785	0.551	1.117	0.178				
Nucleoside analogue or interferon	No	1.000	-	-	-	1.000	-	-	-
	Yes	0.666	0.535	0.831	0.0003	0.593	0.360	0.977	0.040
Initial treatment	RFA	1.000							
	Surgery	0.935	0.783	1.117	0.461				

All statistical tests were two-tailed and used a type I error rate of 0.05 (*p*). AFP: α-fetoprotein; ALT: alanine aminotransferase; ALP, alkaline phosphatase; CI: confidence interval; HBV: HBsAg positive; HCV: anti-HCV antibody; HR: hazard ratio; INR: International Normalized Ratio; RFA: local radiofrequency ablation; SII was defined as follows: neutrophil × platelet/lymphocyte.

## Data Availability

The datasets used and analyzed for this study are available from the corresponding author upon request.
